# Supplementary material for: Development and evaluation of an online HIV pre-exposure prophylaxis (PrEP) training program for community pharmacists to implement pharmacy-led PrEP services in Malaysia
Source: PLoS One. 2025 Aug 18;20(8):e0328713. doi: 10.1371/journal.pone.0328713 (PMC12360552; doi:10.1371/journal.pone.0328713)
Supplement: S1 File — (DOCX) [file pone.0328713.s001.docx]

# **Supplemental Information File 1.**

## **Pre- and post-training knowledge test questionnaire.**

This questionnaire was designed for community pharmacists to complete before and after the online self-paced PrEP training to assess their knowledge of PrEP pre- and post-training.

**This test consists of 20 questions. You are required to complete this test before and after the online training. Read each question carefully and choose the BEST answer. Please answer each question truthfully without the aid of the internet or other resources as the aim of this test is to determine the effectiveness of online training.**

1. What is pre-exposure prophylaxis (PrEP)?

a. An antiretroviral medication used to treat HIV.

**b. An antiretroviral medication used to prevent HIV before possible exposure to HIV.**

c. An antiretroviral medication used to prevent HIV after possible exposure to HIV.

d. A type of medication used to prevent sexually transmitted infections.

2. Which of the following risk behaviour(s) is **NOT** indicative for PrEP?

a. Condomless sex with multiple partners or partners with unknown HIV status.

b. Injecting drug use.

c. Engage in transactional sex.

**d. Condomless sex with an HIV-positive partner who is on HIV treatment and has an undetectable viral load.**

3. What medication is used for PrEP?

**a. Tenofovir disoproxil fumarate (TDF) + emtricitabine (FTC).**

b. Tenofovir disoproxil fumarate (TDF) + emtricitabine (FTC) + Dolutegravir.

c. Tenofovir disoproxil fumarate (TDF) + emtricitabine (FTC) + Raltegravir.

d. Tenofovir disoproxil fumarate (TDF) + emtricitabine (FTC) + Efavirenz.

4. Which of the following statements about the use of PrEP is **FALSE**?

a. PrEP is safe for pregnant or breastfeeding women.

b. PrEP protects against HIV 1 & 2.

**c. PrEP must be taken for life.**

d. PrEP cannot prevent sexually transmitted infections other than HIV.

5. Who should not be initiated on PrEP?

i. Individuals currently on HIV treatment

ii. Individuals who have eGFR <60 mL/min per 1.73m^2^.

iii. Transgender women on hormonal therapy.

iv. Cisgender women on oral contraceptives.

a. i.

**b. i & ii.**

c. i, ii & iii.

d. ii, iii & iv.

6. Which of the following statements about PrEP effectiveness is **FALSE**?

**a. PrEP reduces the risk of getting HIV from sex and injecting drug use by 99%.**

b. PrEP reduces the risk of getting HIV from sex among MSM by 99%.

c. PrEP reduces the risk of getting HIV from sex among heterosexuals by 99%.

b. PrEP reduces the risk of getting HIV among injecting drug users by 74%.

7. For cisgender women, transgender women on estradiol-based hormones and injecting drug users who are on **daily PrEP**, how long does it take to achieve protective concentration after starting PrEP?

a. Immediately.

b. After 24 hours of daily use.

**c. After 7 days of daily use.**

d. After 30 days of daily use.

8. Which of the following is **NOT** a common side effect of PrEP?

a. Nausea.

b. Headache.

c. Abdominal cramps.

**d. Weight gain.**

9. What is the recommended **duration** of PrEP use for at-risk individuals?

a. 1 month.

b. 6 months.

**c. Indefinitely (during at-risk period).**

d. Life-long.

10. Which of the following are the **additional tests that may be considered before starting PrEP?**

i. Bone mineral density test

ii. Renal profile.

iii. STI screening.

iv. Hepatitis B and C.

a. i

b. ii & iii

**c. ii, iii & iv**

d. i, ii, iii & iv

11. What is the recommended follow-up schedule for continuing PrEP users?

a. Every month.

**b. Every 3 to 6 months.**

c. Every 12 months.

d. As needed.

12. How soon should PrEP users be screened for HIV after the first follow-up?

a. Every month.

**b.** **Every 3 months.**

c. Every 6 months.

d. Every 12 months.

13. Which of the following individuals may be offered **event-driven PrEP**?

i. 19-year-old sexually active cisgender man.

ii. 25-year-old sexually active transgender woman currently taking estradiol-based hormones.

iii. 30-year-old sexually active cisgender man currently injecting drugs.

iv. 35-year-old sexually active transgender woman not taking estradiol-based hormones.

a. i

b. i & ii

**c. i & iv**

d. ii & iii

14. What is the **dosing regimen** for **event-driven PrEP** for a newly initiated PrEP user?

a. 2 pills within 72 hours, and 1 pill 12 hours after the first dose.

b. 2 pills within 72 hours, and 1 pill 24 hours after the first dose.

c. 1 pills 2-24 hours before sex, 1 pill 24 hours after the first dose, and 1 pill 24 hours after the second dose.

**d. 2 pills 2-24 hours before sex, 1 pill 24 hours after the first dose, and 1 pill 24 hours after the second dose.**

15. Which of the following risk factors increases the risk of **renal toxicity** in relation to PrEP use?

i. Older than 50 years old.

ii. Comorbidities such as diabetes, hypertension.

iii. High-dose NSAIDS.

iv. Use of creatine-containing bodybuilding supplements as it may interfere with kidney function test results.

a. i

b. i & ii

**c. i, ii & iii**

d. i, ii, iii & iv

16. What are the **counselling key points** that should be covered for newly initiated PrEP users?

i. Dosing regimen.

ii. Adherence.

iii. Side effects.

iv. Post-exposure prophylaxis (PEP).

a. i

b. i & ii

c. i, ii & iii

**d. i, ii, iii & iv**

17. Which of the following are the **PrEP adherence counselling points**?

i. Take the medication at the same time every day.

ii. Setting phone reminders or alarms.

iii. Use a pill box, adherence diary or app to keep track of pills taken.

iv. Incorporate it into daily activities.

a. i

b. i & ii

c. i, ii & iii

**d. i, ii, iii & iv**

18. What should a person do if the person **misses a dose of daily PrEP**?

a. Take a double dose of PrEP pills to make up for the missed dose.

**b. Take the missed dose as soon as you remember it. However, if it is almost time for the next dose, skip the missed dose and continue your regular dosing schedule.**

c. Skip the missed dose and continue with your regular dosing schedule.

d. Stop taking PrEP altogether.

19. Which of the following is a common reason **why someone** **might stop taking PrEP**?

i. Side effects.

ii. Cost.

iii. Reduced need for PrEP (no longer at-risk).

iv. Difficulty remembering to take it.

a. i

b. i & ii

c. i, ii & iii

**d. i, ii, iii & iv**

20. You encounter a 55-year-old cisgender male client who has been in a monogamous sexual relationship with an HIV-negative man for the past 2 years. Should PrEP be discussed with this client?

**a. No, because he is in a monogamous relationship with an HIV-negative individual.**

b. No, because he is older than 50 years of age.

c. Yes, but only if he is not using condoms.

d. Yes, because he is a sexually active man who has sex with men.
