# Supplementary material for: Development and evaluation of an online HIV pre-exposure prophylaxis (PrEP) training program for community pharmacists to implement pharmacy-led PrEP services in Malaysia
Source: PLoS One. 2025 Aug 18;20(8):e0328713. doi: 10.1371/journal.pone.0328713 (PMC12360552; doi:10.1371/journal.pone.0328713)
Supplement: S2 File — (DOCX) [file pone.0328713.s002.docx]

# **Supplemental Information File 2.**

## **Post-training feedback form.**

This is a feedback form for community pharmacists who have completed the online training to provide feedback on the online training received.

**Please tell us what you think about this online training so that we can improve it in the future.**

1. Please indicate your level of agreement with the following statements.

| No. | Item | Strongly disagree | Disagree | Neither agree nor disagree | Agree | Strongly agree |
| --- | --- | --- | --- | --- | --- | --- |
| 1. | The training covered the content I expected. |  |  |  |  |  |
| 2. | The content was organized and easy to follow. |  |  |  |  |  |
| 3. | The content was easy to understand. |  |  |  |  |  |
| 4. | The duration of training was appropriate. |  |  |  |  |  |
| 5. | The additional resources shared were helpful. |  |  |  |  |  |
| 6. | Overall, I am satisfied with this training. |  |  |  |  |  |
| 7. | This training will be useful for my work. |  |  |  |  |  |
| 8. | I would recommend this to others. |  |  |  |  |  |

2. What aspects of this online training program were most useful or valuable?

………………………………………………………………..

3. How would you improve this online training program?

………………………………………………………………..

4. Do you have other comments or questions?

………………………………………………………………..
