# Supplementary material for: Development and evaluation of an online HIV pre-exposure prophylaxis (PrEP) training program for community pharmacists to implement pharmacy-led PrEP services in Malaysia
Source: PLoS One. 2025 Aug 18;20(8):e0328713. doi: 10.1371/journal.pone.0328713 (PMC12360552; doi:10.1371/journal.pone.0328713)
Supplement: Attachment — Submitted filename: Response to Reviewers.docx [file pone.0328713.s004.docx]

**Dr. Rafdzah Ahmad Zaki**
Public Health Medicine Specialist and Professor,

Department of Social and Preventive Medicine,

Faculty of Medicine
Universiti Malaya, Malaysia

rafdzah@ummc.edu.my

25^th^ April 2025

**Editor-in-Chief
PLOS ONE**

**Subject: Rebuttal Letter for Manuscript Revision (PONE-D-24-57343)**

Dear Dr. Ali Ahmed,

We sincerely appreciate the opportunity to revise our manuscript, titled "Development and delivery of an online HIV pre-exposure prophylaxis (PrEP) training for community pharmacists to prepare for the implementation of a pharmacy-led PrEP service in Malaysia" (Manuscript ID: PONE-D-24-57343).

We are grateful for the constructive feedback provided by the reviewers and the academic editor. We have carefully addressed each of the comments and have made the necessary revisions to enhance the clarity and quality of our manuscript. Below, we provide a point-by-point response to each comment, detailing the changes made.

# Journal requirements:

**Comment:** Please ensure that your manuscript meets PLOS ONE's style requirements, including those for file naming. The PLOS ONE style templates can be found at

https://journals.plos.org/plosone/s/file?id=wjVg/PLOSOne_formatting_sample_main_body.pdf and https://journals.plos.org/plosone/s/file?id=ba62/PLOSOne_formatting_sample_title_authors_affiliations.pdf.

**Response:** We have reviewed and updated our manuscript to ensure compliance with PLOS ONE’s style requirements, including formatting and file naming conventions. The revised manuscript follows the guidelines provided in the PLOS ONE style templates.

**Comment:** Please upload a copy of Supplemental Information File 1 and Supplemental Information File 2 to which you refer in your text on page 9. Please amend the file type to 'Supporting Information'. If the Supplementary file is no longer to be included as part of the submission, please remove all reference to it within the text.

**Response:** We have uploaded Supplemental Information File 1 and Supplemental Information File 2 as 'Supporting Information' files, as per the journal’s requirements. It was initially included in the manuscript under S1 Appendix and S2 Appendix. Additionally, we have ensured that all references to these files within the manuscript are accurate.

# Response to the Academic Editor’s Comments:

**Comment:** I have only concern of sample diversity as most of participants are Chinese, given the diversity of Malaysia. Does this impact the implications of your findings?

**Response:** We acknowledge this concern and have addressed it in the manuscript. The predominance of Chinese pharmacists in our sample reflects the demographic distribution in private pharmacies in Malaysia. However, the training program was adapted from local PrEP training for general practitioners and follows local guidelines and core PrEP competencies applicable to all pharmacists. Future efforts will focus on broader outreach to enhance diversity in participation.

# Response to Reviewer 1’s Comments:

**Comment 1:** Consider revising the title - Development and delivery of an online HIV pre-exposure prophylaxis (PrEP) training for community pharmacists to prepare for the implementation of a pharmacy-led PrEP service in Malaysia. This title could be made more explicit e.g., Development and Evaluation of an Online PrEP Training Program for Community Pharmacists to Enhance Pharmacy-Led HIV Prevention Services in Malaysia.

**Response:** We agree and revised the title to “Development and evaluation of an online HIV pre-exposure prophylaxis (PrEP) training program for community pharmacists to implement pharmacy-led PrEP services in Malaysia”.

**Comment 2:** Abstract - The phrases "stigmatized key populations" and "non-traditional differentiated service delivery models, like pharmacies" are slightly repetitive when describing the rationale. Simplify for conciseness. While informative, the committee, platform, and timeline description could be condensed. Focus on the core aspects, such as the training content, evaluation tools, and key methodological steps. While the results highlight the improvement in knowledge scores, the discussion of gaps in knowledge and variability among participants is not explored sufficiently. Briefly mention specific gaps to better contextualize the need for enhanced training methods. Link participant demographics to their potential impact on training outcomes. While the conclusion mentions the need for modern methodologies, it does not connect how these would address the identified gaps.

**Response:** The abstract has been refined to enhance clarity and remove redundant phrases while maintaining key information on training content, evaluation, and methodological aspects.

**Comment 3:** Introduction - While the introduction thoroughly describes the HIV epidemic in Malaysia, key affected populations, and existing service delivery gaps, it is overly detailed, with some points repeated unnecessarily, e.g., barriers to public healthcare facilities and the potential of pharmacy-based models. The transition between some paragraphs feels abrupt, particularly when discussing global recommendations, local implementation, and the rationale for pharmacy-led models. The introduction outlines the problem well but doesn't sufficiently emphasize how the study addresses these gaps.

**Response:** We have streamlined the introduction to reduce redundancy and tightened the narrative, particularly around barriers to existing service delivery and the rationale for pharmacy-based models. Transitions between paragraphs have been improved to ensure a more coherent flow from global recommendations to local implementation efforts. We have also revised the final paragraph to more explicitly state how our study addresses the identified gaps by developing and piloting an online PrEP training module specifically for community pharmacists—an essential step toward enabling future pharmacy-led PrEP service delivery in Malaysia.

**Comment 4:** The Materials and Methods section provides detailed descriptions of the study's design, processes, and tools, which is commendable. The EPIS framework adds rigor and aligns the study within a recognized implementation science framework, strengthening the study's credibility. However, the section is densely packed with information, making it challenging to follow. Key components like "Development of the training program," "Delivery," and "Data analysis" could be more distinct. Breaking the section into clearly labeled subsections, e.g., Training Program Development, Study Design, Participant Recruitment, Training Delivery, Evaluation, and Data Analysis, would improve readability. Certain details, e.g., repeated mentions of module content and pre/post-test descriptions, are unnecessarily repeated. While the stakeholder consultation is mentioned, its contributions to the training program development are not elaborated upon. The author should highlight specific insights or recommendations from stakeholders that informed the program design to strengthen the relevance of the consultation. There is insufficient explanation of why particular tools, e.g., Edpuzzle and Google Forms, were chosen. The section uses long paragraphs that make key details hard to locate.

**Response:** We agree and have included subsections to improve clarity and reduced redundancy. The use of Edpuzzle and Google Forms are not necessarily salient here, but we include them to ensure that readers understand which methods we selected – mostly for ease when interacting with participants. We do, however, provide more explanation for how these platforms work and why we used them.

**Comment 5:** Results -The results section contains substantial data organized around demographic characteristics, pre- and post-training knowledge scores, feedback on the training program, and participant suggestions for improvement. Demographic information is detailed but repetitive, with percentages mentioned in both text and table form. Summarize key demographic trends in the text and refer readers to the table for further details. Table 3 presents data participant-by-participant, which may not be necessary for interpretation. Condense Table 3 to highlight summary statistics (mean, SD, minimum, maximum, p-value) without individual scores. The narrative describing pre- and post-training knowledge scores is overly general and does not critically engage with the data. For example, the statement “the online training appears insufficient” is unsupported by nuanced analysis. Provide a more detailed interpretation of the data. The feedback is summarized well but lacks a deeper synthesis or connection to broader findings. Authors need to analyze feedback to identify recurring themes, e.g., clarity of clinical content, need for quizzes, the pacing of modules, etc, and link these themes to observed knowledge gaps.

**Response:** We appreciate the reviewer’s detailed feedback and have revised the Results section accordingly. To reduce redundancy, we have summarized key demographic trends in the text while referring readers to the table for details. In Table 3, we have added a column showing the percentage change in scores and the overall percentage change across participants. We believe retaining this table in the main manuscript is important to illustrate individual pre- and post-training scores, especially since four participants did not show improvement after training. Additionally, we have refined the discussion of knowledge scores to provide a more critical interpretation of the data, ensuring conclusions are supported by nuanced analysis.

To provide a more critical engagement with the data and to address the reviewer’s suggestion to link participant demographics to training outcomes, we conducted additional statistical analyses that were not part of the original manuscript. Specifically, independent t-tests were used to examine differences in post-training knowledge scores by gender (male vs. female), education level (undergraduate vs. postgraduate), and prior HIV-related training (yes vs. no), while one-way ANOVA was used to compare scores across age groups, ethnicities, and years of professional experience. These analyses are now described in the Data Analysis subsection of the Methods, with results reported briefly in the Results section. No statistically significant differences were found across these demographic variables, suggesting that the online training program was broadly effective regardless of participants’ background characteristics. However, this finding should be interpreted with caution due to the small sample size, which we have acknowledged as a limitation in the revised manuscript.

The statement on training sufficiency has been revised for accuracy. We have also expanded the synthesis of participant feedback, identifying recurring themes such as clarity of clinical content, the need for interactive elements (e.g., quizzes), and module pacing, linking these to observed knowledge gaps for a more comprehensive interpretation.

**Comment 6:** Discussion section—While improving knowledge scores is significant, the discussion could delve deeper into how these scores translate into practical application or readiness to provide PrEP services. The discussion briefly mentions areas where the training fell short, e.g., indications, side effects, and contraindications, but it does not explore why these gaps persist or propose specific ways to address them in future iterations. While participant feedback is mentioned, the discussion does not thoroughly analyze or contextualize it. For example, the perceived disconnect between participants’ satisfaction and actual knowledge gains could be explored in more depth. The discussion does not sufficiently address potential barriers to implementing the training program on a larger scale, such as resource constraints, internet accessibility, or variations in pharmacists' baseline knowledge. While the study's relevance to public health goals is noted, e.g., achieving zero new HIV infections in Malaysia by 2030, the practical steps needed to integrate pharmacists into the national PrEP service framework are not discussed. Although the ERIC taxonomy is cited, its application in this context is not detailed. Explaining how ERIC strategies could specifically enhance pharmacist training would strengthen the argument.

**Response:** We agree and have expanded the Discussion section to better contextualize how improved knowledge scores translate into pharmacists' readiness to provide PrEP services. We have explored persistent knowledge gaps, particularly in indications, side effects, and contraindications, and proposed strategies such as case-based learning. Additionally, we have noted that in-person training included a review and discussion of questions with lower correct response rates. We have also analysed the disconnect between participant satisfaction and actual knowledge gains and expanded discussions on barriers to scaling up the training, including resource constraints and baseline knowledge variations. To strengthen relevance to national public health goals, we have outlined practical steps for integrating pharmacists into Malaysia’s PrEP service framework, including policy support, pharmacist certification pathways, and collaboration with healthcare providers. Finally, we have elaborated on the application of ERIC strategies that might be useful to enhance implementation.

**Comment 7:** Conclusions—The conclusions summarize the study's key findings, emphasizing the novelty and effectiveness of the online PrEP training program in increasing pharmacists' knowledge. However, there is an overgeneralization. For example, while the training program improved knowledge, the conclusions imply broader success without fully addressing the uneven knowledge gains among participants or the remaining critical knowledge gaps. Recommendations for incorporating advanced learning methodologies are mentioned but lack specificity. For example, how case-based or simulation-based learning could be practically integrated is not explored. The conclusions do not discuss potential logistical or systemic challenges, e.g., funding, time constraints, or accessibility issues that might hinder nationwide program implementation. There is little mention of how increased pharmacist knowledge translates into actual improvements in PrEP service delivery or uptake in Malaysia. While knowledge improvement is highlighted, confidence and skill in counseling and service provision are not sufficiently addressed, even though they are critical for successful PrEP delivery.

**Response:** We agree and have revised the Conclusions section to avoid over-generalization and ensure a more balanced interpretation of the findings. We now acknowledge the variability in knowledge gains among participants and highlight remaining critical knowledge gaps. Additionally, we have specified how active learning methodologies, such as case-based and simulation-based learning, could be practically integrated into future training to enhance the development of applied skills. We have also included a discussion on potential logistical and systemic challenges, such as funding, time constraints, and accessibility issues, that may affect nationwide implementation. While this study focused on improving pharmacists’ knowledge as a foundational step toward implementation readiness, we have acknowledged in the limitations that we did not assess confidence or counselling skills directly. We have clarified in the conclusions section that future research should explore how knowledge improvement translates into real-world counselling skills, confidence, and effective PrEP service delivery outcomes.

# Response to Reviewer 2’s Comments:

Congratulations to the authors for the well written manuscript. The manuscript highlights a salient issue on the development and delivery of online PrEP training for new cadres such as pharmacy providers in the context of task shifting services. Two issues for consideration:

**Comment 1:** The title of the manuscript gives prominence to the development and delivery of the online training. However, the primary substance of the manuscript is on PrEP knowledge gains from undertaking the training. Would the authors consider reframing their title to address the primary focus of their manuscript.

**Response:** We have revised the title – please see response to Reviewer 1.

**Comment 2:** The limitations of the study are well described but no strengths are included. It would be good to read why the authors think this research adds to the field and why their study is a strong one.

**Response:** A section discussing study strengths, including its novel approach to pharmacist training and alignment with implementation science frameworks, has been added.

# Response to Reviewer 3’s Comments:

This manuscript focuses on development and assessment of an online, self-paced PrEP training program for community pharmacist for PrEP delivery in Malaysia. This is the first known assessment of such a training program in Malaysia and adds to the sparse literature on how pharmacy providers could be trained to deliver PrEP services in Malaysia and other global settings. While addressing an important topic, some further details on how the training was developed and what went into development of the assessment tools would be helpful. Additionally, it isn’t clear why the authors choose to only describe the online component of this training program and not the in-person training that followed—which seemed to utilize many of the training strategies the authors suggest for enhancement of the online training module. I think presenting both training components together would enhance this paper and present a wholistic package of how pharmacy providers might be trained on PrEP delivery. Further, both the Introduction and Discussion of the paper could be revised for clarity and focus.

## Major:

**Comment 1:** (Abstract, Results): It isn’t clear how to interpret the “knowledge score”. How was this constructed and scored; what types of questions were included in this assessment?

**Response:** We have clarified the methodology used to develop the knowledge assessment, including the construction of question items, scoring, and interpretation of knowledge scores. The full set of assessment questions can be found in Supplemental Information File 1.

**Comment 2:** (Abstract, Results): It is not totally clear what you mean from the following sentence: “While training significantly improved knowledge scores, it did not produce consistent knowledge gains for all participants or address some critical PrEP knowledge gaps”. Can you be more specific on the gaps of interest? And how many participants didn’t have knowledge gains?

**Response:** For the abstract, we have revised to be more specific about the knowledge gaps and number of participants who didn’t have knowledge gains. We have further elaborated on specific knowledge gaps, particularly in counselling key points, identifying PrEP candidates, contraindications, effectiveness, and management of missed doses for daily PrEP in the Results section, and how these gaps inform future training iterations in the Discussion section.

To further explore the variability in knowledge gains and assess whether participant characteristics influenced post-training knowledge scores, we conducted additional statistical analyses not included in the original submission. Independent t-tests and one-way ANOVA were used to compare post-training scores across gender, education level, prior HIV-related training, age groups, ethnicity, and years of professional experience. These results, now summarized in the Methods and Results sections, showed no statistically significant differences, suggesting that participant characteristics did not systematically account for variations in knowledge outcomes. However, this finding should be interpreted cautiously due to the small sample size.

**Comment 3:** (Abstract, Conclusions): Might temper conclusions because not proving in this abstract that pharmacy PrEP delivery increases PrEP access.

**Response:** We agree and have revised the Conclusions section in the abstract to ensure that it does not overstate the impact of pharmacy-led PrEP delivery on PrEP access. We have reworded the statement to clarify that while pharmacy-led PrEP services have the potential to expand PrEP availability, this study did not directly assess its impact on PrEP uptake. Additionally, we have refined the conclusion to focus on the knowledge gains achieved through the training while emphasizing the need for further enhancements and larger studies to assess pharmacist confidence, counseling skills, and service implementation outcomes.

**Comment 4:** (Introduction): The introduction could be abbreviated for focus and clarity.

**Response:** We agree and have revised the Introduction to improve focus and clarity.

**Comment 5:** (Methods): How was the content of the open-ended questions analyzed? Which subsequently led to key themes on the most useful aspects of the training and areas for improvement presented in the Results?

**Response:** We agree and have clarified in the Methods section that open-ended responses were summarized descriptively to highlight key insights on the most useful aspects of the training and areas for improvement. Responses were grouped by common topics, with all unique responses included, while overlapping responses were excluded.

**Comment 6:** (Discussion, paragraph 2): Still not clear where your suggested threshold of 80% knowledge comes from. Seems arbitrary to suggest such threshold if different knowledge assessments using different tools.

**Response:** We appreciate the reviewer’s comment and have clarified the basis for the 80% knowledge threshold in the Discussion section. This threshold was informed by a literature review of studies assessing pharmacists’ PrEP knowledge in various settings, including Utah (61%), Zimbabwe (58%), and U.S. pharmacy students (78.8%). While no study has established a formal cut-off score for PrEP competency, we proposed that pharmacists undergoing PrEP training should ideally achieve a knowledge score of at least 80%, exceeding the baseline knowledge levels of pharmacy students. We have revised both the methods and discussion to explicitly state this rationale and acknowledge that further research is needed to establish an evidence-based competency threshold for PrEP knowledge. We also provide further justification for this cutoff in the methods section.

**Comment 7:** (Methods): Details on how pharmacists were trained on HIV testing required for PrEP delivery missing. Especially since this might require in-person practice that is difficult to delivery remotely.

**Response:** The pharmacy-led PrEP service involves pharmacists facilitating HIV self-testing, which is user-friendly and largely self-explanatory – HIV self-testing is a manualized and evidence-informed and easy testing practice. Pharmacists independently learned the process through instructional videos and leaflets provided to them. However, in-person training was required for communicating test results and managing participants’ reactions, especially for reactive cases. We have added a brief explanation that this was covered in the in-person workshop, which included guidance on effective communication, emotional support, encouraging confirmatory testing, and collaboration with NGOs for participant follow-up and seamless referral to care.

**Comment 8:** (Discussion): Unclear what you would measure the effect of training on? If using as an ERIC implementation strategy, wouldn’t the ultimate outcome be PrEP uptake at the pharmacy versus knowledge among providers? And you did see a positive change in knowledge among providers post-training, so unclear why you are suggesting that the training wasn’t effective at knowledge gain?

**Response:** We appreciate the reviewer’s comment and have clarified this point in the Discussion section. The primary objective of the training was to provide the required knowledge, and thus, its effect was measured by the change in knowledge scores. This is a pre-implementation activity and is important in the preparation phase of implementation. Though the scores did increase, the scores remained low still for several participants. The reference to ERIC implementation strategies was to emphasize that online training alone is not sufficient for successful implementation and that a mix of strategies—such as case-based, problem-based, simulation-based, peer-assisted, team-based, and interactive learning—could further enhance training effectiveness. While we conducted an in-person workshop to improve hands-on and communication skills, our shortfall was not bundling the entire training (including the workshop) and assessing knowledge levels post-workshop. We have added this as one of our limitations. Our suggestion that training was not fully effective for knowledge gain was based on the observation that four participants did not show improvement in knowledge scores, which we have now described in greater detail in the Discussion section.

**Comment 9:** (Discussion): You note that modern learning methodologies (e.g., case-based, team-based) could improve knowledge outcomes among pharmacy providers – in the methods, might be worth mentioning why you didn’t incorporate any of these approaches when you developed this online training.

**Response:** We have addressed why modern learning methodologies were not integrated into this iteration of the online training and highlighted how future iterations could incorporate case-based and team-based learning approaches to enhance knowledge retention.

**Comment 10:** (Discussion): IF the online training was just part one of provider training, followed by the in-person training – why is the in-person training not included in this manuscript to complete the discussion of how you might train pharmacy providers for PrEP delivery?

**Response:** We have clarified that this manuscript focuses solely on the online training component as a standalone activity designed to equip community pharmacists with the foundational knowledge to deliver PrEP services. The in-person training was intended to complement this by focusing on hands-on practice and communication skills. To maintain a focused discussion, the in-person training component was excluded from this manuscript. However, we acknowledge that assessing knowledge levels after the in-person training would have provided additional insights, as the case-based discussions included in that phase could have further enhanced participants' knowledge.

**Comment 11:** (Discussion & limitations): These sections of the paper are long and could be abbreviated for focus and clarity.

**Response:** The Discussion section has been streamlined to enhance clarity while maintaining key findings and interpretations.

## Minor:

**Comment 1:** (Formatting): Formatting of the current manuscript is a little confusing – the distinction between sections and subsections is not clear. Additional subsections (e.g., data collection, analysis) in the methods could enhance clarity.

**Response:** We agree and have added additional subsections to improve clarity, particularly in the Methods section, by clearly distinguishing sections such as training development, training delivery, evaluation and data analysis.

**Comment 2:** (Methods): The April 2023 stakeholder meeting is mentioned without details on what types of stakeholders attended this meeting and what was discussed. Adding a brief description of this would be helpful.

**Response:** We have provided details on the April 2023 stakeholder meeting, including the types of stakeholders involved and key insights that informed training development, including feedback on module content and competencies.

**Comment 3:** (Methods, Delivery of the online self-paced PrEP training program): Would suggest moving several details here (e.g., who attended the training, duration of engagement) to the Results section.

**Response:** Certain details from the training program delivery section have been moved to the Results section for better alignment and clarity.

**Comment 4:** (Results, Table 3): Not sure inclusion of this table in the main paper is necessary – would suggest including as an appendix, if want to keep.

**Response:** We have added the column that describes the change in score (%) and overall percentage change across participants. We believe it is important to retain this table in the main manuscript to illustrate individual pre- and post-training scores, as well as score changes, particularly since four participants did not show any improvement in knowledge after training. See further response to Reviewer 1.

**Comment 5:** (Results, Table 4): Would consider adding a column that is the % change from pre- to post-training assessment. Could include overall score across question at bottom of this table (i.e., info in Table 3).

**Response:** We have added the column that describes the percentage change and overall score across question at the bottom of the table.

We hope that our revisions adequately address the concerns raised. Please let us know if further modifications are required. Once again, we appreciate the reviewers’ and editor’s valuable feedback, which has helped us improve our manuscript.

Thank you for your time and consideration.

Best regards,

**Dr. Rafdzah Ahmad Zaki**

Public Health Medicine Specialist and Professor,

Department of Social and Preventive Medicine,

Faculty of Medicine

Universiti Malaya, Malaysia
rafdzah@ummc.edu.my
