# Supplementary material for: Development and evaluation of an online HIV pre-exposure prophylaxis (PrEP) training program for community pharmacists to implement pharmacy-led PrEP services in Malaysia
Source: PLoS One. 2025 Aug 18;20(8):e0328713. doi: 10.1371/journal.pone.0328713 (PMC12360552; doi:10.1371/journal.pone.0328713)
Supplement: Attachment — Submitted filename: Response to Reviewers_3 July 2025.docx [file pone.0328713.s005.docx]

**Dr. Rafdzah Ahmad Zaki**
Public Health Medicine Specialist and Professor,

Department of Social and Preventive Medicine,

Faculty of Medicine
Universiti Malaya, Malaysia

rafdzah@ummc.edu.my

25^th^ June 2025

**Editor-in-Chief
PLOS ONE**

**Subject: Rebuttal Letter for Manuscript Revision [PONE-D-24-57343R1] - [EMID:7ee32a2b256e6417]**

Dear Dr. Ali Ahmed,

We sincerely appreciate the opportunity to revise our manuscript, titled "Development and evaluation of an online HIV pre-exposure prophylaxis (PrEP) training program for community pharmacists to implement pharmacy-led PrEP services in Malaysia." (Manuscript ID: PONE-D-24-57343R1).

We are grateful for the constructive feedback provided by the reviewer. We have carefully addressed each of the comment and have made the necessary revisions to enhance the clarity and quality of our manuscript. Below, we provide a point-by-point response to each comment, detailing the changes made.

# Journal requirements:

**Comment:** Please review your reference list to ensure that it is complete and correct. If you have cited papers that have been retracted, please include the rationale for doing so in the manuscript text or remove these references and replace them with relevant current references. Any changes to the reference list should be mentioned in the rebuttal letter that accompanies your revised manuscript. If you need to cite a retracted article, indicate the article’s retracted status in the References list and also include a citation and full reference for the retraction notice.

**Response:** We have carefully reviewed the reference list to ensure its completeness and accuracy. All citations were cross-checked, and no retracted articles were identified. Therefore, no rationale for including retracted references was necessary. Minor formatting corrections were also made where appropriate, and these are reflected in the revised manuscript.

The reference below has been added to replace the previously cited newspaper articles, as the official statistics on the number of PrEP users as well as the number of government clinics providing PrEP services are now available in the Malaysian Global AIDS Monitoring Report 2024.

3. Ministry of Health Malaysia. 2024 Global AIDS Monitoring: Country Progress Report - Malaysia. Putrajaya; Ministry of Health Malaysia; 2024.

~~3. Ministry of Health Malaysia. 2023 Global AIDS Monitoring: Country Progress Report - Malaysia. Putrajaya: Ministry of Health Malaysia; 2023.~~

~~8. Vethasalam R, Tan T, Gimino G, Lee B. More than 2000 individuals given PrEP to reduce HIV infections, says Dr Zaliha. Star [Internet]. 2023 Nov 7 [cited 2024 Mar 22]; Available from: https://www.thestar.com.my/news/nation/2023/11/07/more-than-2000-individuals-given-prep-to-reduce-hiv-infections-says-dr-zaliha~~

~~11. Ibrahim J, Rahim R, Lai A, Pfordten D. Ten more govt clinics to start dispensing HIV prevention drugs, says Dr Dzulkefly. Star [Internet]. 2024 Mar 20 [cited 2024 Mar 22]; Available from: https://www.thestar.com.my/news/nation/2024/03/20/ten-more-govt-clinics-to-start-dispensing-hiv-prevention-drugs-says-dr-dzulkefly~~

Additionally, please find below the references that were added in the first revision but were not mentioned in the previous rebuttal letter.

31. McKeirnan K, Czapinski J, Bertsch T, Buchman C, Akers J. Training Student Pharmacists to Perform Point-of-Care Testing. American Journal of Pharmaceutical Education. 2019;83(7):7031.

32. Alzahrani MM, Alghamdi AA, Alghamdi SA, Alotaibi RK. Knowledge and Attitude of Dentists Towards Obstructive Sleep Apnea. International Dental Journal. 2022;72(3):315–21.

41. Loh P, Chua SS, Karuppannan M. The extent and barriers in providing pharmaceutical care services by community pharmacists in Malaysia: a cross-sectional study. BMC Health Services Research. 2021;21:1–14.

# Response to Reviewer 4’s Comments:

**Comment 1:** Line 106: Can you explain what you mean by strategic task shifting?

**Response:** We have revised the sentence to clarify the meaning of strategic task-shifting as follows:

"Furthermore, community pharmacists are well-trained healthcare professionals and well-positioned to provide PrEP services through strategic task-shifting, which involves the appropriate reallocation of responsibilities such as counselling and dispensing from physicians to pharmacists to improve service delivery and access."

**Comment 2:** What modifications were made while adapting the physician-targeted training program for pharmacists?

**Response:** We have clarified this in the revised manuscript under “training program development”. While adapting the physician-targeted HIV Connect PrEP course for pharmacists, we simplified certain content to focus more on oral PrEP (tenofovir disoproxil fumarate/emtricitabine), rather than options that were either unavailable or not approved for PrEP in Malaysia at the time, such as tenofovir alafenamide/emtricitabine (TAF/FTC), long-acting injectable cabotegravir, and the dapivirine ring. A summary of special circumstances was added to aid comprehension. Additionally, implementation-focused videos were incorporated to provide practical guidance on key aspects of the pharmacy-led PrEP service, including checklists for PrEP initiation and follow-up, adherence assessment and counselling, and referral pathways (when and who to refer). These modifications were made to align the training with pharmacists’ scope of practice and enhance implementation readiness.

**Comment 3:** Line 402: The word ‘cisgender’ may need explanation.

**Response:** We appreciate the reviewer’s comment. “Cisgender” is a commonly used term in public health literature and reflects the terminology used in the cited article. However, to improve clarity and consistency, we have revised the term to “cisgender men who have sex with men (MSM)” in the manuscript.

**Comment 4:** Can more details about the in-person workshop be provided?

**Response:** Thank you for this suggestion. As the primary focus of this manuscript is the development and evaluation of the online PrEP training program, we have kept the description of the in-person workshop concise in the Materials and methods section to avoid shifting focus. However, additional details about the workshop have been included in the Discussion section (lines 481 to 494; and lines 544 to 546) to provide further context on its role as a supplementary reinforcement activity.

**Comment 5:** Lines 486 to 489. A sentence is repeated.

**Response:** The repeated sentence has been removed.

We hope that our revisions adequately address the concerns raised. Please let us know if further modifications are required. Once again, we appreciate the reviewer’s and editor’s valuable feedback, which has helped us improve our manuscript.

Thank you for your time and consideration.

Best regards,

**Dr. Rafdzah Ahmad Zaki**

Public Health Medicine Specialist and Professor,

Department of Social and Preventive Medicine,

Faculty of Medicine

Universiti Malaya, Malaysia
rafdzah@ummc.edu.my
